# Scent Goes Digital: The Role of Insect Odorant Binding Proteins in Modern Technology

**DOI:** 10.1002/biof.70066

**Published:** 2026-01-19

**Authors:** Maddalena Ventura, Miriam Viola, Krishna C. Persaud, Antonio Guerrieri, Carmen Scieuzo, Patrizia Falabella

**Affiliations:** ^1^ Department of Basic and Applied Sciences University of Basilicata Potenza Italy; ^2^ Department of Biology University of Naples Federico II Napoli Italy; ^3^ Department of Chemical Engineering and Analytical Science The University of Manchester Manchester UK; ^4^ Spinoff XFlies S.R.L., University of Basilicata Potenza Italy

**Keywords:** biological electronic nose, biomimetic, biosensor, insects, odorant binding proteins, volatile organic compounds

## Abstract

Researchers have developed hybrid bionic platforms for odor detection, inspired by natural chemoreceptive systems, advancing artificial olfactory systems that recognize specific volatile compounds. Odorant binding proteins (OBPs) are small carrier proteins found in the olfactory organs of mammals and insects. When coupled with electrical transducers, OBPs act as recognition elements, converting chemical signals into electrical outputs. This enables the development of biological electronic noses that are based on biomimetics and aim for sustainability. The objective of this review is to provide a comprehensive and updated overview of OBP‐based biosensors, with a particular focus on insect OBPs as biorecognition elements, and to critically examine their applications, advantages, and technological potential across different fields. OBP‐based biosensors show strong promise in medical diagnostics, environmental monitoring, food quality, insect pest control, and security. Insects demonstrate remarkable sensitivity to specific odors which makes them excellent models for designing bioinspired biosensors. Compared to conventional methods, OBP‐based biosensors offer significant advantages in terms of portability, rapid response, and cost‐effectiveness. OBPs are remarkably stable under different environmental conditions and can bind both volatile and aqueous‐phase molecules, enhancing their functional versatility. Moreover, they can be produced through biotechnological processes using renewable resources, supporting eco‐friendly innovation. These advantages make OBPs ideal candidates for next‐generation biosensors in fields requiring real‐time and on‐site chemical detection.

## Graphical abstract

This review delves into bioinspired olfactory biosensors, focusing on insect odorant binding proteins (OBPs) as core elements of hybrid bionic platforms for volatile compound detection. Highlighting the development of the biological electronic nose (BE‐NOSE), it examines current prototypes, outlines advantages over conventional methods, and explores applications in diagnostics, environmental monitoring, food safety, pest control, and security.

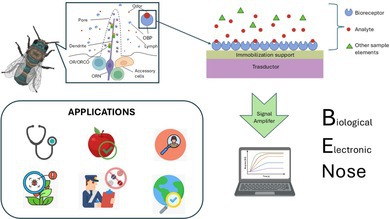

## 1 General Information on Biosensors

The ability of chemosensory systems to assess the chemical composition of the environment to detect beneficial or harmful conditions and objects has inspired the development of platforms that mimic or improve upon these natural processes [1]. Safeguarding ecosystems from exploitation and degradation has become a global priority. In this context, the sustainable use of natural principles and processes positions biomimetics and biological transformation among the leading frontiers of the so‐called “Fourth Industrial Revolution” (Industry 4.0). Byrne et al. define “Biologicalisation in Manufacturing” as “the use and integration of biological and bio‐inspired principles, materials, functions, structures, and resources for intelligent and sustainable production technologies and systems with the aim of reaching their full potential” [2]. Similarly, Miehe et al. emphasize the importance of a convergence between industrial technologies and biological processes, promoting a circular economy that utilizes renewable resources and biomaterials derived from natural cycles, thereby reducing environmental impact and resource consumption [3]. Simultaneously, the emergence of nano‐bioelectronics, a multidisciplinary field that integrates nanomaterials and nanoscience with biology and electronics, has overcome existing bioelectronic challenges and opened new frontiers [4]. From this perspective, the concept of engineering the olfactory system of living organisms has surfaced, creating an artificial olfactory system capable of detecting and distinguishing volatile chemical substances in a gas mixture to identify specific conditions [1]. The incorporation of bio‐inspired technologies into chemical detection and analysis systems presents remarkable opportunities for applications in areas such as medical diagnostics, environmental monitoring, and food quality control, as they emulate the efficiency and versatility inherent in natural biological systems.

Recent reviews and studies [5, 6, 7] have emphasized how biomimetic strategies, particularly in olfaction‐inspired biosensors, can improve analytical platforms through enhanced sensitivity, miniaturization, and multi‐sensor integration. Among other applications, chemosensors are employed in quality control processes during food production [8, 9] as well as in environmental monitoring, with particular attention to the detection of toxic or harmful compounds [10, 11]. There are also extensive applications in agriculture, where these devices can perform environmental and soil monitoring, pesticide detection, and facilitate waste management [12]. Furthermore, these sensors can also be utilized to support security operations, including the detection of explosives, chemical weapons, or smuggling [13]. Advanced devices of this type represent a significant step toward an industrial future guided by the principles of biomimetics and sustainability. In the biomedical field, chemosensors can detect volatile organic compounds (VOCs) emitted by a patient—the so‐called volatilome—and serve as tools to monitor the metabolic state of a patient or to non‐invasively diagnose pathological conditions such as cancer [14, 15, 16, 17, 18, 19, 20].

This review provides a comprehensive analysis of odorant binding proteins (OBPs), small soluble proteins capable of binding and transporting odorant molecules [21], with a particular emphasis on insect‐derived OBPs, and explores their potential as biorecognition elements in advanced biosensors. It outlines the developmental prospects for a biological electronic nose (BE‐NOSE) that leverages insect OBPs to selectively identify specific gaseous compounds through target‐molecule recognition. To achieve this, the review also examines the behavioral aspects of insect olfaction, offering insights into how these natural models can complement molecular studies of OBPs. This integrated perspective enhances our understanding of OBP functionality and also informs the design of bioinspired biosensors for detecting specific chemical compounds. Finally, several case studies are presented to illustrate the performance and applicability of insect OBP‐based biosensor prototypes, highlighting current achievements and identifying promising opportunities for further development in this emerging field.

### 1.1 Overview of Biosensor Technologies and Bioinspired Recognition Elements

The concept of the “electronic nose” (E‐NOSE) was introduced in the 1980s, with the first prototype based on a sensor array developed by Persaud and Dodd [22]. The objective of an intelligent bioelectronic device is to identify odors, which are mixtures of chemical substances, rather than discriminate single molecules, associating them with specific conditions.

Currently, solid‐phase microextraction gas chromatography–mass spectrometry (SPME‐GC/MS) is typically used to analyze and identify odor molecules [23] as well as the headspace gas chromatography–mass spectrometry (HS‐GC/MS); it plays an important role in routine analysis [24]. While SPME‐GC/MS offers high precision, it also has significant disadvantages, including multi‐step procedures, long analytical times, large instrumentation, and the need for highly qualified personnel and expensive reagents [25]. In response to these limitations, biosensors emerge as a solution that significantly improves accessibility, practicality, and cost‐effectiveness in the detection of selected analytes across a wide range of applications while maintaining the same degree of efficiency.

Several commercial E‐NOSEs have been developed with applications in many industrial fields, such as food and beverage, agriculture, healthcare, environmental monitoring, and manufacturing. These devices, commonly based on materials such as metal oxides or conductive polymers [26, 27, 28, 29, 30], exploit the principle that the adsorption of odor vapors on semiconductor surfaces or polymer films leads to a variation in electrical resistance (conductance), thus allowing for the quantification of the concentrations of the compounds of interest. However, these coating materials cannot faithfully reproduce the biological characteristics of the olfactory system, limiting their accuracy in odor detection [31].

Given that the olfactory systems of many living organisms can discriminate a wide variety of odor molecules, the use of olfactory biological materials could enable the development of an olfactory biosensor that more closely mimics the sensitivity and specificity of these natural systems [32], characteristics particularly useful for the detection of VOCs at low concentrations [33, 34, 35].

The organization of the biosensor, as described by Bhalla et al. [36], includes several key elements. The bioreceptor, a specific molecule capable of recognizing the analyte, plays a fundamental role in the bio‐recognition process, generating a signal upon interaction with the analyte. The transducer then converts this biochemical event into a measurable physical signal, usually optical or electrical, which reflects the strength of the interaction between the analyte and the bioreceptor. The electronic section processes the transduced signal through complex circuits and algorithms that process the data, allowing for amplification and conversion from analogue to digital. Finally, the processed signals are quantified by the biosensor display unit, providing a clear and comprehensible interpretation of the obtained data.

Most currently available biosensors have been developed by integrating bioreceptors with various transducers [37, 38]. Among these are devices that measure mass vibrations per unit area to detect various types of gases, such as quartz crystal microbalances (QCM) [39, 40], and surface acoustic wave (SAW) sensors [41]. Other transducers include EIS [42, 43], diamond bio‐microelectromechanical systems (MEMS) [44, 45], and field‐effect transistors (FET) [46]. All these advanced detection systems allow for the detection and quantification of the interaction between the analyte and the recognition element, enabling a translation of the chemical signal into a quantifiable electrical signal. The qualitative data, however, depend on the affinity of the materials used for detection toward the target molecules. The ability to detect volatile ligands at biologically relevant concentrations is crucial for the expansion of the emerging field of bio‐detection [47]. Incorporating components inspired by natural biological systems can provide a level of sensitivity comparable to that observed in nature.BE‐NOSEs can rapidly recognize selected target molecules and can be used more simply than conventional SPME‐GC/MS methods, which require pre‐treatments and qualified technicians. Moreover, E‐NOSEs can be integrated and multiplexed into small chips, making them portable and suitable for on‐site analysis. Therefore, a BE‐NOSE may be more suitable for some complex applications compared to traditional GC/MS approaches [48].

The technological development of biosensors is advancing rapidly. Prototypes have been designed and developed using sensitive biological materials for olfactory detection, such as whole cells or even tissues like insect antennae, olfactory receptors (ORs), OBPs, and synthetic polypeptides derived from various living organisms, both vertebrates and invertebrates [34, 49, 50, 51, 52, 53, 54].

ORs, being G protein‐coupled transmembrane proteins, are difficult to stabilize outside the cellular environment, limiting their use in biosensors, as they require specific conditions to maintain their structure and functionality. In contrast, OBPs offer numerous advantages: they are easily synthesized in heterologous systems, stable over a wide range of temperatures, resistant to proteolytic degradation, and functional even in the presence of organic solvents. They exhibit remarkable stability when exposed to air, which makes them highly suitable for biosensor applications without requiring stringent environmental regulation, an advantage not shared by ORs [55, 56].

**FIGURE 1:**
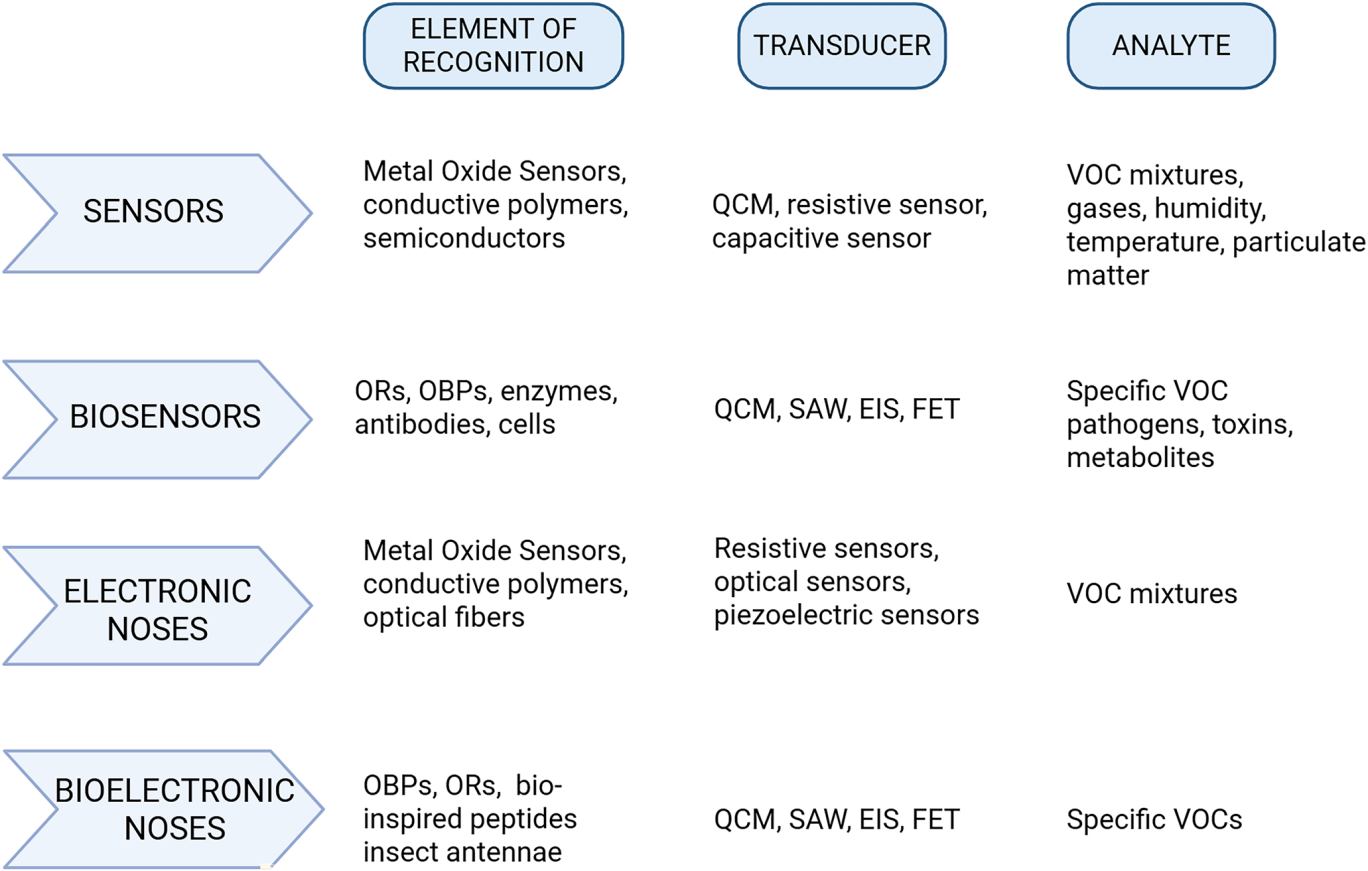
Graphical comparative schema illustrating the main differences among traditional sensors, biosensors, electronic noses, and bioelectronic noses in terms of recognition elements, transducer type, and detectable materials.

However, the development of E‐ and BE‐NOSEs still faces significant challenges. The main limitations are the complexity of olfactory coding and the difficulty of reproducing the high sensitivity of biological systems. In mammals, only about 50 of the 300–400 ORs have been de‐orphanized, and new strategies are required to overcome current sensitivity constraints [6]. In E‐NOSEs, analytical accuracy is compromised by several factors, including diminished sensitivity caused by water vapor or elevated analyte concentrations, sensor drift over time, and the absence of absolute calibration standards [57]. Moreover, the absence of specific regulations and standardization complicates comparison and validation among devices (Figure 1), limiting their broader industrial use.

## 2 Chemoreception in Insects

Chemoreception refers to the capacity to detect specific chemical cues and is among the most ancient mechanisms by which organisms engage with their environment. This sensory process plays a vital role in interpreting environmental stimuli, enabling the detection of food, alerting to dangers or predators, and facilitating social communication [58]. Throughout the life cycle of insects, chemoreception is crucial, as it responds to a variety of chemical, biological, and environmental signals. This mechanism assists in finding and choosing food sources, mates, places for laying eggs, and evading predators [59, 60]. Insect chemosensory systems detect a wide range of volatile and soluble chemicals and play a crucial role in all of the contexts described above [60].

Chemosensory neurons reside within specialized sensory organs known as sensilla. In numerous insect species, olfactory sensilla are primarily situated on two key head structures, the antennae, and maxillary palps. Conversely, gustatory sensilla are scattered across various body regions, such as the internal and external mouthparts, legs, wing edges, and, in females, the ovipositor [60]. The insect sensillum, derived from the epidermis, is a specialized hair‐like or peg‐like structure that protrudes from the antennae and functions as a receptor for chemical and physical stimuli in the surrounding environment. Olfactory sensilla are characterized by numerous pores in the cuticle, allowing semiochemicals to pass through and interact with receptors on the dendritic membrane [61].

In insects, the detection of chemicals is facilitated by molecules identified as olfactory, gustatory, and ionotropic receptors, along with soluble olfactory proteins like OBPs and chemosensory proteins (CSPs). OBPs and CSPs are particularly important in carrying hydrophobic chemical compounds from the external environment to the sensillar lymph, where they are then delivered to receptors on the membranes of sensory neurons [59, 62]. In addition to OBPs and CSPs, Niemann–Pick type C2 (NPC2) proteins represent a newly recognized class of soluble proteins involved in insect chemoreception, functionally complementing OBPs and CSPs. These small proteins belong to the ML family (MD‐2‐related lipid recognition) and are characterized by a compact β‐sheet structure that confers affinity for lipophilic molecules and semiochemicals [63]. Similar to insect OBPs, NPC2 proteins exhibit a conserved motif of six cysteines paired in three disulfide bonds [64]. Their role in olfactory processes was first demonstrated in the ant *Camponotus japonicus* , where an NPC2 protein was localized in the antennal chemosensilla and identified as a mediator of chemical communication [65]. In arthropods, NPC2s are encoded by multigene and highly divergent families, in contrast to vertebrates, which possess a single NPC2 gene mainly associated with cholesterol transport [63, 66]. This gene expansion is considered an evolutionary adaptation to chemical communication, suggesting that NPC2s act as carrier proteins for semiochemicals and participate in olfactory signal transduction [67]. Experimental findings have reinforced the involvement of these proteins in olfactory processes: NPC2 has been identified in the antennal sensilla of *Helicoverpa armigera*, while gene silencing in *Varroa destructor* disrupted host detection and altered behavioral patterns. In both vertebrates and insects, these proteins are present at high concentrations, undergo rapid turnover, and bind a wide array of hydrophobic ligands, typically with micromolar dissociation constants. Moreover, a single species can express multiple OBP isoforms, each exhibiting distinct sequences, and specialized functions that enable selective interaction with various odorant molecule classes [64, 68, 69].

### 2.1 Insect Odorant Binding Protein

The term OBP refers to two structurally distinct protein classes: vertebrate OBPs, belonging to the lipocalin superfamily and folded into a β‐barrel motif [70, 71, 72], and insect OBPs, mainly composed of α‐helical domains [21]. Despite their structural differences, both classes share a similar function [64]. OBPs are primarily associated with olfaction and chemoreception, being present in the sensory organs of both vertebrates and insects, particularly abundant in the nasal mucus of vertebrates and the sensillar lymph of insects [73]. OBPs were first identified almost simultaneously in mammals [74] and in insects [75]. The first insect OBP, discovered in the giant moth *Antheraea polyphemus* [75] was named pheromone‐binding protein (PBP) for its affinity for sex pheromones [73]. Thousands of OBPs have been discovered through genome sequencing, and hundreds have been functionally characterized using fluorescent ligand‐binding assays. Twenty‐one functional studies have examined 382 distinct OBPs from 91 insect species, culminating in the development of iOBPdb, a database classifying OBP binding affinities for 622 different VOC targets [76].

Insect OBPs are small proteins consisting of 130–150 amino acids (13–17 kDa) [60], but can be grouped into long‐, medium‐, and short‐chain types, based on their amino acid length [77, 78]. OBPs share a pattern of six cysteines, the relative positions of which are well conserved across all insect orders. Based on the cysteine pattern, OBPs can be divided into different categories, including classical OBPs with six positional conserved cysteines, paired into three interlocked disulfide bridges, following a specific motif pattern (C_1_‐X_25‐30_‐C_2_‐X_3_‐C_3_‐X_36‐42_‐C_4_‐X_8‐14_‐C_5_‐X_8_‐C_6_) [79], plus OBPs, with more than six cysteines [80], minus‐OBPs, with less than six cysteines, [81] and atypical OBPs, with more than eight cysteines [59, 80, 81, 82]. This cysteine pattern has become a “signature” for classical insect OBPs [73]. The presence of a conserved pattern of six cysteines and three disulfide bridges limits the flexibility of the molecule but ensures a greater resistance to degradation and denaturation [58]. These characteristics prevent thermal denaturation and attack by proteolytic enzymes [83]. Structurally, OBPs consist of six α‐helical domains forming a compact fold with a hydrophobic cavity designed to bind and transport odorant molecules [21]. They display remarkable thermal and pH stability, and can refold to their native conformation after denaturation, retaining their functional integrity [42, 84]. Another group of small soluble polypeptides in insects is CSPs, found in the olfactory and gustatory organs. These proteins are highly expressed in the lymph of chemosensilla and display binding activity toward odorants and pheromones [69, 73, 85, 86, 87, 88]. CSPs are around 100–120 residues long and present a conserved pattern of four cysteines forming two disulfide‐linked loops and consist of α‐helical domains arranged in a folding distinct from that of OBPs [87, 89, 90, 91]. OBPs are essential for detecting environmental cues and mediating key behaviors like host identification, mate selection, predator avoidance, and oviposition [78]. When volatile compounds come into contact with an insect's antennae, they enter through countless tiny pores on the outer surface of the sensilla and diffuse into the sensillar lymph, a watery medium rich in soluble proteins like CSPs, sensory neuron membrane proteins (SNMPs), and odorant‐degrading enzymes (ODEs). These proteins are essential for odor detection and work in tandem with odorant and ionotropic receptors (IRs) to facilitate signal transmission within the olfactory system [92, 93, 94].

**FIGURE 2:**
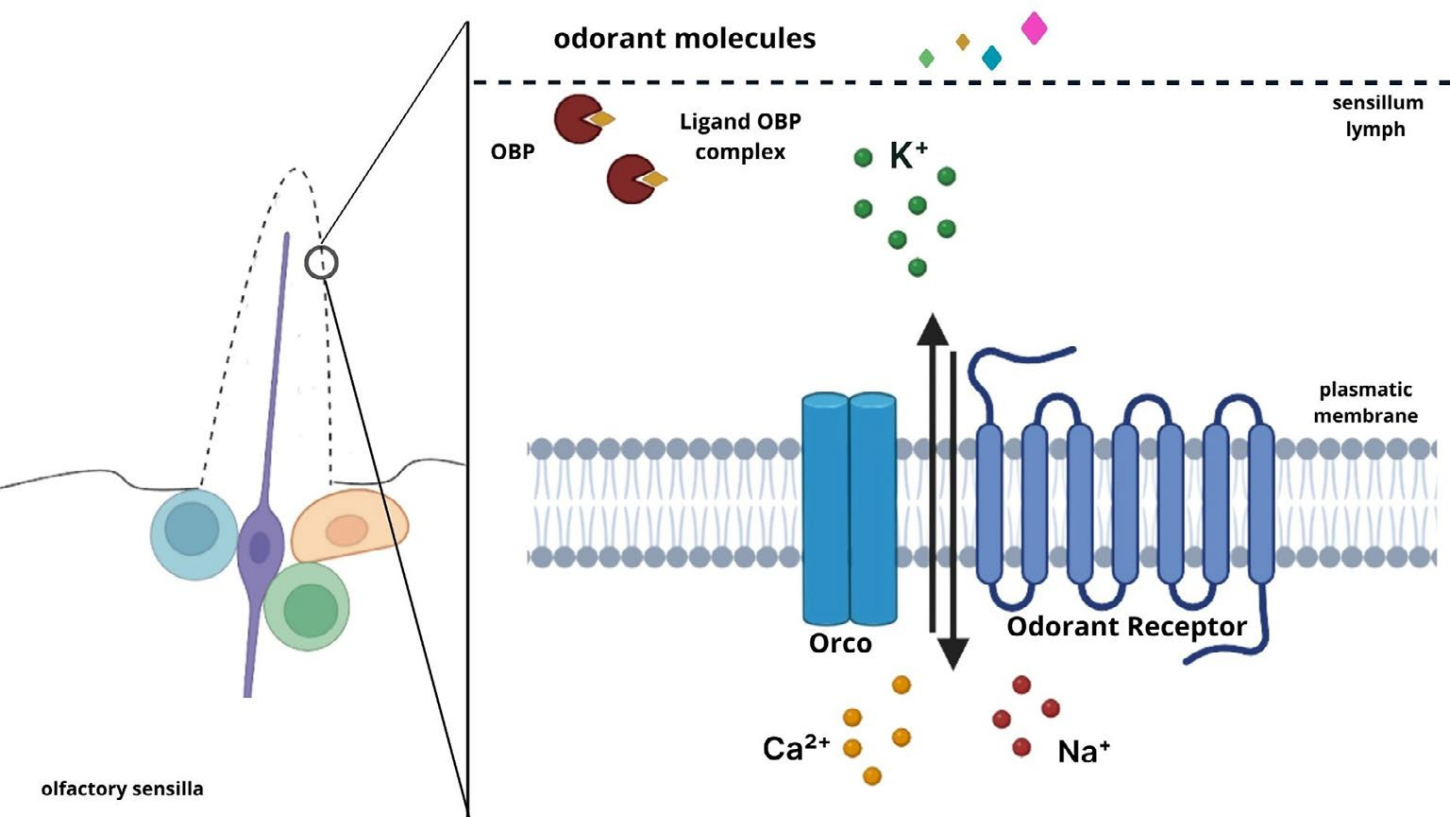
Schematic of the olfactory process in insects: Odor molecules pass through sensillar pores, bind to OBPs, are transported to ORs, and activate the signaling cascade leading to odor perception.

Odor molecules diffuse through sensillar pores and are captured by OBPs, which transport them across the sensillar lymph to ORs located on the membranes of sensory dendrites. Unlike vertebrate G protein‐coupled receptors, insect ORs function as ligand‐gated ion channels activated upon odorant binding [95]. Two major receptor families mediate this process: ORs and IRs, both forming heteromeric odor‐gated ion channels [95]. Each olfactory sensory neuron generally expresses one or a few ligand‐binding ORs alongside the highly conserved co‐receptor Orco, which is essential for proper receptor assembly and signal transmission [96, 97, 98]. ORs typically feature seven transmembrane domains (TMDs) with diverse extracellular regions that enable recognition of a wide range of ligands [96, 97]. The absence of Orco, first discovered in *Drosophila melanogaster* , leads to impaired receptor trafficking and a loss of olfactory‐driven behaviors [99, 100, 101, 102, 103]. The odor‐OBP complex initiates receptor activation, which may occur via two mechanisms: a single receptor responding to multiple odorants or a single odorant activating multiple receptors [104, 105, 106, 107]. The ligand‐gated ion channel formed by the insect OR complex, together with OBPs and other sensillar lymph proteins, constitutes a highly specialized system enabling efficient environmental chemical detection [108] (Figure 2).

### 2.2 Insect OBPs Versus Mammalian OBPs

In mammals, OBPs and PBPs are acidic polypeptides of 150–160 amino acids 17–20 kDa [83], present in their native state as non‐covalent dimers or monomers. Based on their amino acid sequence and three‐dimensional structure, these proteins can be classified as part of the lipocalin superfamily [109, 110, 111]. They serve a dual function: they solubilize and transport volatile pheromones to ORs and bind these molecules in specialized glands to facilitate their release into the environment [112]. These protein families are expressed in mammalian body fluids, such as urine and saliva, which are known to contain species‐specific pheromones [73, 113]. All OBPs and PBPs share with other lipocalins the typical β‐barrel three‐dimensional fold, a cup‐shaped cavity formed by eight antiparallel β‐strands, complemented by a short α‐helix segment near the C‐terminus [110]. Lipocalins also share a short ‐G‐X‐W‐ motif located near the N‐terminus, which is fully conserved. Sequence identity among distant members of the lipocalin family can be very low, even below 20%. Despite this high variability, the tertiary structure is well‐conserved, with the typical β‐barrel motif formed by eight antiparallel β‐sheets [109, 111]. Cysteine residues also play a significant role in the stability of vertebrate OBPs [114]. OBPs of vertebrates and insects do not exhibit homology in their amino acid sequence nor similarity in their three‐dimensional structure [21, 115, 116], although they share the function of capturing, transporting, and releasing small VOCs with broad specificity within a micromolar range, through the fluid surrounding sensory dendrites [117, 118].

Insect OBPs, owing to their unique structure and intrinsic properties, represent an ideal solution for robust and cost‐effective detection devices (Table 1).

**TABLE 1:** Comparison of vertebrate and insect OBPs: The table compares insect and vertebrate OBPs in terms of their physicochemical, functional, and structural characteristics, highlighting differences in structure, stability, biological function, and biotechnological applications.

Characteristics	Vertebrate OBPs	Insect OBPs
Protein structure	β‐barrel composed of eight antiparallel β‐sheets and a short α‐helical segment	Compact structure with six α‐helical domains
Protein length	150–160 amino acids	120–150 amino acids
Stability	Stable, with limited contribution from cysteines	High stability due to conserved cysteine disulfide bridges
Cysteine pattern	Sometimes absent (e.g., in bovines)	Six conserved cysteines forming three disulfide bridges
Primary function	Transport and release of odorants	Binding with volatile hydrophobic molecules for communication
Biotechnological applications	Environmental monitoring, odor release systems	Robust and sensitive detection devices for odorants
Immunological behavior	Potential role in innate immunity	Not reported
Distribution between sexes	Present in both sexes	Classified into PBP and GOBP: pheromone‐specific in males (PBP) or general (GOBP)

Olfactory stimuli are composed of volatile molecules with a wide range of chemical structures. Natural scents are intricate blends, where both the composition and relative concentrations of components shape their unique identity. Even seemingly simple odors are challenging to characterize and measure, as perception is influenced by multiple molecular attributes, such as size, shape, and functional groups, rather than a single parameter like wavelength [119, 120]. Structural differences, including isomerism and chirality, can also lead to distinct olfactory experiences [6]. To interpret this complexity, the human olfactory system utilizes over 300 receptors [121, 122], a modest number compared to the vast array of odorant molecules in the environment. It distinguishes millions of scents through a combinatorial coding approach, wherein each receptor can respond to multiple odorants, and each odorant can activate several receptors to varying extents [123, 124, 125]. Similarly, *D. melanogaster* has developed a highly effective olfactory system, capable of detecting and differentiating a broad spectrum of odorants using only about 60 odorant receptor genes. These genes are expressed in subsets of approximately 1300 olfactory neurons within its antennae, employing the same combinatorial coding strategy [126].

Functional analyses have shown that its OBPs also follow a combinatorial recognition pattern: suppression of single OBP genes alters behavioral responses to multiple odorants, and conversely, several OBPs can modulate the detection of the same odorant molecule [127]. This highlights a double‐layered olfactory coding strategy in insects, in which combinatorial activation of OBPs precedes receptor activation, effectively enhancing odor discrimination [127].

One of the key challenges in replicating biological olfaction lies in achieving its extraordinary sensitivity. The “olfactory threshold,” the lowest concentration detectable by an average individual, varies significantly across compounds, ranging from parts per million (ppm) to parts per trillion (ppt), spanning several orders of magnitude [6]. Accurately determining the number of odorant molecules that reach the nose is complicated by environmental variables. In certain instances, mere picograms can suffice to elicit an olfactory response. Insects, however, exhibit even greater sensitivity, especially to pheromones. For instance, studies estimate that just 0.396 femtograms of bombykol, ~1000 molecules, striking the antenna of the silkworm moth *Bombyx mori* are enough to provoke a behavioral reaction to this species‐specific sex pheromone [128, 129, 130].

## 3 Olfactory Behavioral Analysis of Insects: Implications for Biomarker Detection and Biosensor Applications

The primary goal for many developers of portable devices is to replicate the extraordinary capabilities of biological olfactory systems, creating tools with detection and discrimination abilities similar to those achieved by biological sensory elements. Observing and analyzing the olfactory behaviors of insects provides critical insights into detection mechanisms, facilitating the selection of species attracted to characteristic odors of specific contexts or conditions. Such attraction can stem from either intrinsic evolutionary components, which cause insects to respond to certain odors through natural adaptation, or from targeted training that enables the recognition of specific odors, supported by the insects' olfactory selectivity toward the molecules under investigation.

Since these behaviors derive from the transduction of complex molecular events, in which OBPs constitute the first molecular level of VOC recognition, this causal relationship provides a solid rational basis for the identification of high‐performance OBPs and their use as recognition elements in advanced biosensors.

Studies, for example, show that infection by *Plasmodium falciparum*, responsible for malaria, alters the odor profile of the human host, inducing the production of specific volatile compounds that increase attraction in *Anopheles gambiae* mosquitoes, which are malaria vectors [131, 132, 133]. These findings suggest that parasite‐induced odor changes could be exploited for developing new malaria control strategies, including diagnostic tools based on volatile biomarkers and traps enhanced by specific odor cues. Ants of the species *Formica fusca* have proven to be promising tools for cancer detection through the perception of VOCs. In experiments on human cancer cells and mice xenografted with human tumors, ants were trained to distinguish the odor of cancer cells or the urine of sick mice from healthy samples. Although the VOC mixture in question may not be ecologically relevant to ants, it still elicits a response, indicating that ants can discriminate among complex odors. This suggests that *F. fusca* may possess OBPs with affinity for these compounds [134, 135].

In Strauch et al. [136], the potential of the olfactory system of the fruit fly ( *D. melanogaster* ) to detect VOCs produced by cancer cells was investigated. Using calcium imaging techniques, researchers monitored neural activity on the flies' antenna surfaces when exposed to odors from cancerous and healthy cells. Specific variants of the OR in *D. melanogaster* , such as dOR43a, dOR45b, dOR7a, and dOR85f, respond to benzaldehyde [137], which shows potential as a biomarker for ventilator‐associated pneumonia (VAP) caused by *Staphylococcus aureus* and *Pseudomonas aeruginosa* [138]. The genetic removal of these receptors reduces the response to this compound, eliminating avoidance behavior.

Another example is heptanal, known both as a biomarker for lung cancer [139] and as a volatile compound emitted by plants. In *Grapholita molesta*, heptanal serves as a significant indicator for food detection, activating the OR GmolOR12 in olfactory neurons on the antennae [140]. Similarly, ORs in *Triatoma infestans* respond to heptanal in a dose‐dependent manner, which is crucial for attraction behavior toward vertebrate odors [141]. In *Aedes aegypti* mosquitoes, heptanal detection facilitates host discrimination, although the specific receptor responsible for this function has yet to be identified [142].

Wasps, particularly the species *Microplitis croceipes* , have proven capable of recognizing and memorizing new chemical signals typically associated with the presence of hosts or food resources, which they use for orientation [143, 144, 145]. This learning process can be replicated in the laboratory through a simple conditioning procedure in which wasps are exposed to a specific odor in association with a food reward. In the presence of the target volatile compound, wasps exhibit specific behaviors such as coiling (a posture adopted when approaching the odor source), active searching, and antenna movement [146, 147, 148]. *M. croceipes* wasps are highly sensitive to 3‐octanone and myrcene. Research findings indicate that wasps have a significantly lower detection threshold for these compounds compared to the Cyranose 320 E‐NOSE. They exhibit 74 times greater sensitivity to 3‐octanone and 94 times greater sensitivity to myrcene, showcasing their remarkable ability to detect very low concentrations of these volatile compounds [149]. Recent research has shown that *M. croceipes* can learn and respond behaviorally to a range of “non‐native” volatile compounds significant to forensic science, such as 2,4‐DNT [150], 3,4‐dinitrotoluene (3,4‐DNT) [144], cyclohexanone [144, 151], methyl benzoate [151], cadaverine, and putrescine [143, 149]. Tomberlin et al. trained wasps to detect 2,4‐DNT, which is a volatile discriminator of trinitrotoluene (TNT) [150]. The conditioned wasps showed a significant response to 2,4‐DNT, particularly to the concentration used during conditioning.

Honeybees, such as the domesticated *Apis mellifera* , possess sophisticated olfactory memory that allows them to remember odors associated with food resources and actively search for these sources in their environment. This behavior has received significant attention over the years [152, 153, 154, 155, 156]. This natural foraging behavior can be leveraged for the detection of volatile odorants, including explosives, by training them to recognize specific odor traces. The studies conducted by Bromenshenk et al. [157] focused exclusively on the ability of honeybees to detect and locate landmines. Their field tests indicated that honeybees could detect vapor compounds at concentration levels from ppb (parts per billion) to ppt (parts per trillion) [157]. Bees can be trained within 1 or 2 days by introducing target odor traces into their feeder, and subsequently, when they leave the hive, they tend to follow the traces of the same odor. This long‐range scouting ability allows them to cover large areas in search of the odor source, with detection efficiency estimated at concentrations as low as a few ppt. However, the flight behavior of bees is influenced by environmental factors such as rain, strong winds, and low temperatures, limiting their operational capacity in unfavorable weather conditions. For instance, bees were able to positively indicate the presence of 2,4‐DNT generated at an estimated vapor concentration of 50–80 ppt [158].

Moths, such as *Manduca sexta* , have also demonstrated the ability to detect explosive compounds [159, 160, 161].

Recent studies have examined the behavioral response of *Philaenus spumarius* to aromatic plants and essential oils [162]. These studies confirmed that *P. spumarius* can perceive a wide range of VOCs, including aliphatic aldehydes, alcohols, esters, ketones, terpenoids, and aromatic compounds [163]. OBPs modulate the response to these VOCs by transporting odors from the olfactory sensilla to neural receptors, thus facilitating discrimination between different host plants and influencing the insect's feeding behavior. This process has direct implications for *P. spumarius* 's ability to locate and select host plants, which is crucial for the transmission of pathogens like *Xylella fastidiosa* .

To better illustrate these differences, Table 2 provides an overview of insect olfactory sensing capabilities, comparing key species, target VOCs, application areas, detection sensitivity, and conditioning or training requirements. This comparative summary highlights innate and learned olfactory capabilities in medical, environmental, and security contexts, underscoring their relevance for biomarker discovery and biosensor development.

**TABLE 2:** Overview of insect olfactory detection abilities: The table summarizes key insect species, target VOCs, application areas, detection sensitivity, and conditioning time, providing a cohesive comparison of their natural and trained olfactory capacities across medical, environmental, and security contexts.

Insect species	Target VOCs	Application area	Detection sensitivity	Conditioning/training time
*Anopheles gambiae*	Volatiles associated with *Plasmodium falciparum* infection	Disease diagnosis/malaria control	Detects odor profile alterations in infected hosts	Not required (innate response)
*Formica fusca*	VOCs from human cancer cells and urine of tumor‐bearing mice	Cancer diagnosis	Discriminates healthy vs. diseased samples	Short olfactory conditioning training
*Drosophila melanogaster*	Benzaldehyde	Medical diagnosis/biomarker for VAP	Strong neural response at low concentrations; reduced after receptor knockout	Not required (innate)
*Caenorhabditis elegans*	Benzaldehyde	Chemoreception model organism	Attracted at low concentrations, avoids high concentrations	Not applicable
*Grapholita molesta*	Heptanal	Food detection/feeding behavior	Activates GmolOR12 receptor; high sensitivity	Not required
*Triatoma infestans*	Heptanal	Vertebrate host detection	Dose‐dependent olfactory response	Not required
*Aedes aegypti*	Heptanal	Human host discrimination	Behavioral response to heptanal; receptor not yet identified	Not required
*Microplitis croceipes*	3‐octanone, myrcene, 2,4‐DNT, 3,4‐DNT, cyclohexanone, methyl benzoate, cadaverine, putrescine	Explosive/forensic compound detection	Up to 94× more sensitive than electronic nose (Cyranose 320)	Few conditioning sessions with food reward
*Apis mellifera*	2,4‐DNT, explosive traces	Explosive detection/environmental monitoring	Detects vapor concentrations down to ppt range	1–2 days of training with conditioned feeding
*Manduca sexta*	TNT and related explosive compounds	Explosive detection	High olfactory sensitivity; odor‐oriented behavior	Not required/short conditioning
*Philaenus spumarius*	Aliphatic aldehydes, alcohols, esters, terpenoids, aromatic VOCs	Chemical ecology/plant pathogen monitoring ( *Xylella fastidiosa* )	Broad VOC perception spectrum	Not required (natural detection)

## 4 Emerging Biosensors for Innovative Applications

**FIGURE 3:**
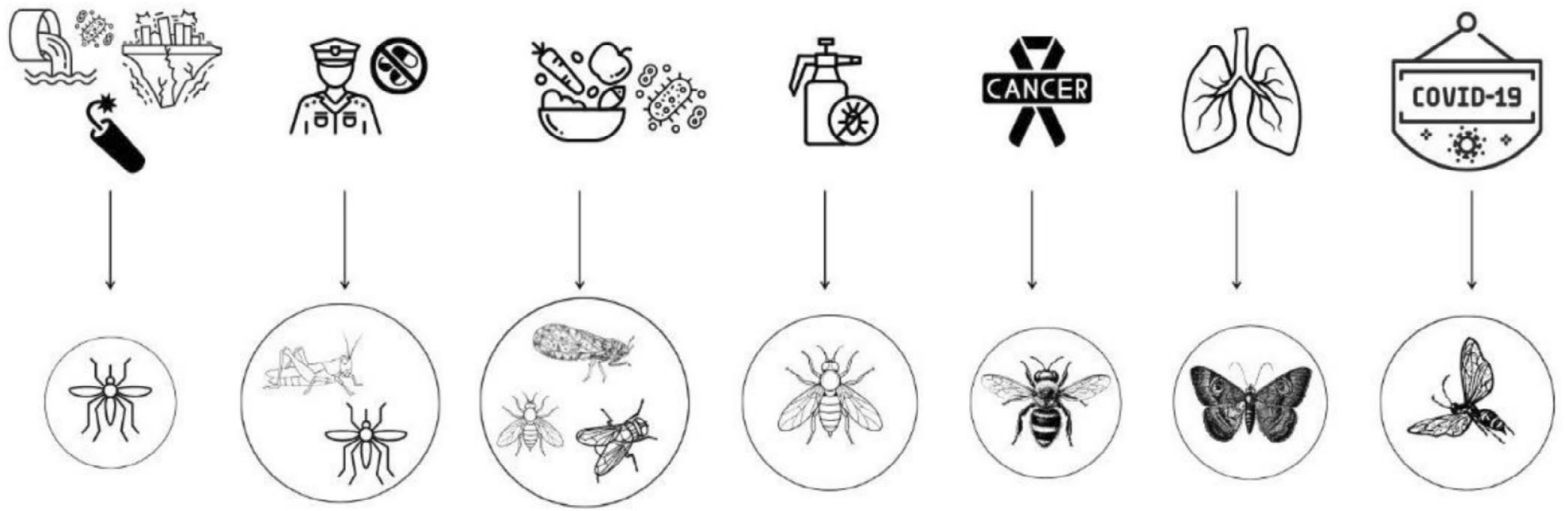
Graphical synthetic illustration of instrument prototypes developed by exploiting insect olfactory systems for the detection of VOCs associated with various application domains, including diseases, environmental contaminants, and hazardous or illicit substances.

Recent advances in biosensor technology have demonstrated the versatility and effectiveness of insect OBPs as bio‐recognition elements for the detection of VOCs with high specificity and sensitivity (Figure 3).

For example, Lu et al. developed an olfactory biosensor based on the antennal‐specific protein of the eastern honeybee *Apis cerana cerana*, the OBP Acer‐ASP2, aimed at the selective detection of floral scents and pheromones using EIS [42]. The Acer‐ASP2 protein, known for its high affinity toward a variety of volatile compounds like pheromones and floral odor, was immobilized on interdigitated gold electrodes. The biosensor responded proportionally to ligand concentration within a detection range of 10^−3^ to 10^−6^ M, confirming sensitivity to compounds present at low concentrations. To better understand protein‐ligand interactions, the authors modeled the three‐dimensional structure of Acer‐ASP2 and used molecular docking to analyze interactions within the hydrophobic binding site of the protein. Docking results showed that several critical amino acid residues participate in molecular recognition, forming specific interactions such as hydrogen bonds and hydrophobic interactions, which explain Acer‐ASP2 high affinity for selected floral scents and pheromones. The developed biosensor represents a promising approach for molecular chemical detection, thanks to the OBP ability to selectively recognize and bind odor molecules even at low concentrations. Acer‐ASP2 properties, including stability and selectivity, demonstrate how insect OBPs can be efficiently used in biosensors for applications like environmental monitoring and air quality control. They provide a concrete example of OBP effectiveness, as their robustness and binding specificity could offer a viable alternative to currently used detection technologies, facilitating the development of portable and selective sensors based on biological components [42].

Shim et al. developed an innovative biosensor based on a bio‐FET with a molybdenum disulfide (MoS_2_) nanopore structure, designed for advanced detection of VOCs through a design that mimics the olfactory system of *D. melanogaster* . The sensor uses the LUSH protein, an OBP from *D. melanogaster* which has a high affinity for 1‐*cis* vaccenyl acetate (VA), its primary ligand [164]. However, LUSH can also bind ethanol, inducing a surface potential change in the bio‐FET, generating indicative electrical signals. This functionality enables the sensor to achieve an ethanol detection limit of 1.71 × 10^−7^ mol/L, with high selectivity and sensitivity, effectively discriminating among various VOCs. The bio‐FET was tested in diverse environments, showing stability even in the presence of glucose, similar to natural conditions in which *D. melanogaster* detects ethanol in fruit. Given the demonstrated sensitivity and selectivity, the authors emphasize its potential in various application fields, such as early medical diagnosis, food quality control, agriculture, and security systems [165].

This section highlights the developments in various fields of innovative applications of OBP‐based biosensors, emphasizing their potential in areas such as environmental monitoring, food safety, pest control, security, and medical diagnostics. These case studies demonstrate how OBPs can serve as robust and selective alternatives to traditional detection methods, fostering the development of portable, efficient, and biologically inspired sensors. To provide a comprehensive overview of these advances, Table 3 summarizes representative OBP‐based biosensors in different application areas.

**TABLE 3:** Summary of OBP‐based biosensors: The table compares sensor designs, target VOCs, detection performance, and main advantages across environmental, food safety, medical, and security applications.

Application area	OBP/protein used	Sensor type	Sensitivity/LOD	Target VOC(s)	Key advantages
Environment (air/floral)	Acer‐ASP2 (*Apis cerana cerana*)	Interdigitated gold electrodes and Electrochemical Impedance Spectroscopy	Detection range 10^−6^–10^−3^ M	Floral odors and pheromones	High selectivity and stability; proportional response; molecular docking confirms binding affinity; suitable for portable air and environmental monitoring sensors
Environment (contaminated water)	AgamOBP1 ( *Anopheles gambiae* )	Portable biosensor (QCM principle)	Detection range 10 ppm‐100 ppb	Coliform‐related analytes	Fast detection compared to culture (24 h); targets coliform metabolites; pocketable
Security (explosives)	AgamOBP4, AgamOBP19 ( *Anopheles gambiae* )	SAW coated with nanodiamonds	Ka = 3.61 μM^−1^ (TNT)	TNT, 2‐amino‐2,4/2,6‐DNT, trinitrobenzene	High affinity/specificity for nitroaromatics; robust SAW platform; effective under low vapor pressures
Security (narcotics)	LmigOBP1 (*Locusta migratoria*)	SAW nanodiamond	Ka = 3.48 μM^−1^ (THC)	THC, cocaine, heroin	Selective recognition of cannabinoids; multi‐analyte capability using the same setup
Security (drugs of abuse)	AgamOBP1_S82P (mutant)	QCM 20 MHz (SAM)	K_d_ = 10^−6^ to K_d_ = 10^−8^ M	Cocaine‐HCl, MDMA, ephedrine	Rationally designed mutant with improved affinity; long‐term operational stability; reusable QCM platform
Victim detection (search and rescue)	pigWTOBP1, AgamOBP1, AgamOBP1_S82P, AgamOBP4	QCM array (on SMURF robot)	Not reported	Living: acetone, n‐heptanal; Deceased: cadaverine, putrescine, skatole, indole	Real‐time discrimination of living vs. deceased; robotic integration; effective under realistic disaster conditions with user interface display
Food safety (bacteria)	Peptide derived from *Drosophila* OBP	CNT‐FET	1fM	VOCs from *Salmonella* (3‐methyl‐1‐butanol, 1‐hexanol)	Ultra‐fast detection; high specificity (no response to healthy/inactive samples); easily integrated into FET devices
Food safety (pesticides)	OBP2 ( *Diaphorina citri* ) + AuNPs	DiNM (LSPR)	LOD: imidacloprid 1.4 ppb, acetamiprid 1.5 ppb, dinotefuran 4.5 ppb	Neonicotinoids in tea (TP = 88%, TN = 100%)	LOD below MRLs; rapid on‐site screening with LC confirmation; low false negatives; multi‐analyte capability
Food quality (decomposition)	HillOBP_C57 ( *Hermetia illucens* )	QCM (SAM on Au)	LOD: 0.013 ppm (isobutyraldehyde), 0.004 ppm (isovaleraldehyde), 0.024 ppm (2‐methylbutyraldehyde)	Aldehydes and acids from decomposition (butyric, isoamyl, etc.)	High sensitivity at room temperature; early‐stage decomposition biomarkers; thermally and chemically stable OBPs
Insect pest control	BdorOBP2 ( *Bactrocera dorsalis* )	PEG‐modified interdigitated electrodes/Electrical Impedance Spectroscopy	Detection range 10^−8^ and 10^−7^ M	Isoamyl acetate, β‐ionone, benzaldehyde	Selective detection of host plant semiochemicals; applicable to targeted pest monitoring and ecological management strategies
Diagnosis (tumors/neuroblastoma)	AmelOBP14 ( *Apis mellifera* )	rGO‐FET (PBSE linker)	K_d_ = 4 μM to K_d_ = 3.3 mM.	Homovanillic acid (HVA)	Quantitative electrical transduction via FET; high selectivity in solution; stable covalent anchoring (PBSE)
Diagnosis (breath)	Peptide from HarmOBP7 (*Helicoverpa armigera*)	QCM (drop‐cast film)	LOD = 1 ppm (RT, 2% ± 1% RH)	Acetaldehyde	OBP‐derived peptide: higher binding site density; better performance vs. MOF sensors at high T; low‐power, room‐temperature operation
Diagnosis VOCs (COVID‐19)	Peptides from MmedCSP3 and BhorOBPm2	CNT‐FET multiplex	ΔI: 1.21 ± 0.43 μA (ethyl butyrate); 0.20 ± 0.09 μA (aldehydes)	VOCs associated with COVID‐19	Multiplexed FET system; rational biomimetic design; rapid and non‐invasive detection
Multi‐sector applications	LUSH ( *Drosophila melanogaster* )	MoS_2_ bio‐FET with nanopore	LOD for ethanol 1.71 × 10^−7^ M	Ethanol, 1‐cis‐vaccenyl acetate (VA)	High sensitivity and selectivity; stable in complex environments (e.g., glucose); scalable for diagnosis, food, agriculture, and security fields

### 4.1 Environment

In the environmental field, OBP‐based biosensors represent a powerful strategy for detecting pollutant‐associated VOCs, such as microbial contamination of drinking water or water intended for domestic use.

In developing countries, access to clean and drinkable water is a significant challenge. Waterborne diseases, resulting from fecal contamination of water resources, pose a serious threat to public health. The ability to effectively monitor local water sources is crucial to ensure water safety; however, there is currently no cost‐effective and reliable method for the rapid detection of contamination. It is essential that even water intended for irrigation and sources not meant for domestic use or direct human consumption meet minimum safety and hygiene standards to protect public health and prevent the spread of diseases caused by pathogenic microbes [166]. A significant indicator organism of contamination in water samples is the bacterium *Escherichia coli* [167]. However, the currently approved methods to detect *E. coli* contamination require a minimum of 24 h [168]. An interesting aspect of many current detection methods is their retrospective nature, which limits the timeliness of results; therefore, the availability of a portable device could more immediately address water safety issues. A field‐friendly biosensor capable of providing rapid results in the case of localized water contamination would be particularly valuable [166].

Biosensors based on the insect OBP AgamOBP1 from *Anopheles gambiae* have been developed as highly specific and sensitive detectors of analytes associated with coliform bacteria [169, 170]. This technology enables rapid identification of *E. coli* contamination even at low levels in water. AgamOBP1 immobilized by conjugation with colloidal gold in a lateral flow device was developed, which exploits the robustness of OBP to offer rapid (< 20 min) and high sensitivity (≈1000× higher than traditional assays) detection of bacterial metabolites in water, paving the way for portable, cost‐effective and suitable platforms for environmental monitoring [166].

### 4.2 Security and Defense

In security applications, the molecular sensitivity of insect OBPs to nitroaromatic and narcotic compounds may represent a valid alternative strategy for the detection of hazardous substances with high sensitivity. The detection and identification of hazardous substances, such as narcotics and explosives, remain a critical challenge for customs and security agencies, particularly due to the low vapor concentrations of these substances and the complexity of the environments in which they are found [171]. These compounds, often concealed in sealed packages or in odor‐rich environments, exhibit extremely low vapor pressures, making them difficult to detect using conventional methods [13]. In recent years, research has focused on developing innovative biosensors based on OBPs to overcome these limitations. These sensors offer robust, selective, and sensitive technology, ideal for monitoring substances in security and control applications.

Scorsone et al. expressed and purified fourteen proteins, including wild‐type and mutant variants of insect and mammalian OBPs [171]. Among the selected insect OBPs were proteins from *Anopheles gambiae* , such as AgamOBP4, AgamOBP5, and AgamOBP19, as well as LmigOBP1 from *Locusta migratoria*. These proteins were expressed in the laboratory without significant genetic modifications and were tested for detecting both explosives and narcotics. The compounds tested included nitroaromatic explosives such as TNT, 2,4‐DNT (dinitrotoluene), 4‐NT (nitrotoluene), 2‐amino‐2,4‐DNT, 2‐amino‐2,6‐DNT, and 1,3,5‐trinitrobenzene, along with ammonium nitrate (NH_4_NO_3_). Additionally, the proteins were tested with narcotics such as tetrahydrocannabinol (THC), cannabinol, cocaine, and heroin.The *in silico* results revealed that the binding pockets of the selected OBPs displayed strong specificity toward nitroaromatic compounds. Specifically, AgamOBP19 demonstrated high affinity for 2‐amino‐2,4‐DNT and 2‐amino‐2,6‐DNT, while AgamOBP5 showed a similar response toward these compounds. Computational simulations further confirmed that the binding energy between ligands and OBPs is a key indicator of affinity, supporting the feasibility of using these proteins in detection devices.Experimentally, some OBPs, selected for their liquid‐phase affinity and stability, were immobilized on SAW biosensors coated with nanodiamonds. Among these, AgamOBP4 stood out for its high affinity toward TNT, characterized by an affinity constant (K_a_) of 3.61 μM^−1^, while AgamOBP19 exhibited excellent results for 2‐amino‐2,4‐DNT, 2‐amino‐2,6‐DNT, and 1,3,5‐trinitrobenzene. LmigOBP1, on the other hand, proved promising for detecting narcotics, showing significant affinity for THC (K_a_ = 3.48 μM^−1^).Of all the proteins tested, AgamOBP4 and AgamOBP19 were particularly effective in detecting nitroaromatic explosives, while LmigOBP1 showed potential for cannabinoid detection.Cali and Persaud explored a novel strategy for creating biosensors that leverage OBPs derived from *Anopheles gambiae* , with the aim of detecting drugs of abuse. The researchers employed a strategy that combines *in silico* modeling, targeted mutagenesis, and experimental characterization. Among the analyzed proteins, AgamOBP1 and AgamOBP47 were compared for their potential in creating stable mutants capable of binding specific target molecules. AgamOBP1 was selected as the optimal model due to its favorable structure and greater adaptability for targeted modifications. The mutations were designed to improve the protein affinity for specific ligands, including cocaine, ephedrine, THC, 3,4‐methylenedioxymethamphetamine (MDMA/Ecstasy), and heroin. The results obtained through in silico modeling demonstrated significant improvements in binding capabilities, which were subsequently confirmed by experimental assays. The wild‐type (WT) proteins and mutants were expressed in *E. coli* , purified, and tested to evaluate their affinity for the selected ligands. The mutant AgamOBP1_S82P showed a particular affinity for cannabinol, MDMA, and cocaine hydrochloride. Subsequently, the mutated proteins were immobilized on 20 MHz QCMs using self‐assembled monolayer (SAM) techniques. The resulting biosensors were exposed to pulses of saturated analyte vapor. The biosensor based on AgamOBP1_S82P demonstrated extremely sensitive and selective detection capability for cocaine‐HCl, maintaining high operational stability for at least 10 months [56]. The expected sensitivity of the OBP‐based biosensor array was estimated to be in the range of 10^−6^–10^−8^ M, consistent with the dissociation constants experimentally determined for the proteins [56].

These findings indicate that OBPs represent an innovative solution for detecting hazardous substances, with potential applications in environmental monitoring, public safety, and customs control. The ability to integrate these proteins into advanced biosensors opens new avenues for improving the precision and sensitivity of detection technologies.

### 4.3 Victim Detection

For search and rescue operations, integrating OBPs into sensing platforms enables the discrimination of human‐related VOCs, aimed at the localization of living and deceased victims in complex environments.

The global community is expected to face a significant increase in natural disasters due to climate change. Strengthening societal resilience against events such as floods, wildfires, and earthquakes has become a priority for many governments. To support rescuers in locating trapped victims, the SMURF robot was developed, featuring a miniaturized detection module (“sniffer”) based on advanced gas and odor sensors. This system identifies gases and vapors emitted by victims, determining their location and status (alive or deceased) through a fuzzy logic algorithm, further enhanced by integrated cameras and microphones to optimize search operations [172]. A biosensor array, based on QCM transducers coated with OBPs, was engineered to detect small odor molecules emitted by victims. The selected proteins (pigWTOBP1, AgamOBP1, AgamOBP1_S82P, and AgamOBP4) exhibit high sensitivity, selectivity, and stability [172]. Among the identified chemical markers for living victims are acetone and n‐heptanal, detected at elevated concentrations in the air and originating from biological sources such as breath, skin, and blood. For deceased victims, the primary markers include cadaverine (pentane‐1,5‐diamine), putrescine (butane‐1,4‐diamine), skatole (3‐methylindole), and indole. Field tests demonstrated the “sniffer” module high efficacy in clearly distinguishing between living and deceased victims. The QCM‐based biosensors produced significant responses to VOCs under realistic scenarios, with signal strength increasing as the robot approached the emission source. For living victims, signals were detected at distances up to four meters, with a marked increase within two meters, corresponding to room entrances or proximity to victims. In rubble scenarios, the detection probability for living victims reached 78%, with data displayed in real time on a dedicated user interface. For deceased victims, the system successfully identified decomposition markers such as cadaverine and putrescine, effectively distinguishing them from emissions of living individuals. This differentiation capability proved critical in reducing false positives and improving the efficiency of rescue operations. These findings confirm the effectiveness of the OBP‐based biosensor integrated into the “sniffer” module in detecting gases and VOCs in complex and realistic contexts. The system has demonstrated its ability to provide reliable real‐time data, significantly enhancing the speed and efficiency of victim search and localization operations, with substantial potential to improve rescue missions during emergency situations.

### 4.4 Food Industry

In the agri‐food sector, biosensors functionalized with OBP can be used for the detection of contaminants, quality assessment, and shelf life of food products.

*Salmonella typhimurium* is a bacterium responsible for food poisoning, commonly caused by the consumption of contaminated foods [173]. Symptoms associated with infection include fever, malaise, abdominal pain, headache, myalgia, nausea, anorexia, and constipation [174]. Various methods are available for effectively detecting *Salmonella* contamination, including enzyme‐linked immunosorbent assays, antibody capture, DNA probes, and PCR analysis [175, 176, 177, 178]. However, conventional methods have low sensitivity, thus limiting the ability to detect pathogens early. Furthermore, GC/MS has been used to analyze VOCs present in contaminated foods, specifically identifying two compounds, 3‐methyl‐1‐butanol and 1‐hexanol, in ham contaminated with *Salmonella* [179]. However, GC/MS is characterized by prolonged analysis times and complex procedures, with high costs [46].

Bioelectronic sensors that use ORs and nanomaterials have been developed for the rapid and simple detection of chemicals. These biosensors mimic the natural mechanism of odor perception, selectively recognizing odor substances. Son et al. designed a new BE‐NOSE to detect *Salmonella* contamination in ham, using a peptide derived from the *Drosophila* OBP in combination with a carbon nanotube field‐effect transistor (CNT‐FET) [46].

To test the biosensor effectiveness in assessing food contamination, fresh and *Salmonella*‐contaminated ham samples were used. The biosensor showed no response to the fresh ham sample but registered a significant signal with a response time of less than 2 s when a contaminated sample was injected. By contrast, non‐functionalized CNTs showed no reactions to the contaminated sample, and inactivated *Salmonella* did not trigger any response. The OBP‐functionalized CNT‐FET exhibited a very low limit of detection (LOD) of 1 fM, confirming its high sensitivity toward *Salmonella*‐associated VOCs. Therefore, the detection of *Salmonella*‐contaminated ham samples was made possible with the OBP‐functionalized CNT channel. The user‐friendliness and speed of a biosensor made it possible to effectively assess *Salmonella* contamination in ham [46].

In the realm of food safety, monitoring chemical residues like pesticides, in addition to detecting biological contaminants, is crucial. Pesticides pose significant threats to both the environment and human health. Notably, neonicotinoid pesticides, some of the most widely used agrochemicals in agriculture to control harmful insects, are strongly linked to these risks [180]. A recent study of Chang et al., developed an innovative method for the simultaneous detection of various neonicotinoid pesticides using a biomimetic sensor that combines the recombinant OBP2 protein from *Diaphorina citri* , functionalized with gold nanoparticles (AuNPs), with digital nanoplasmonometry (DiNM) technology based on localized surface plasmon resonance (LSPR). The sensor was designed to simultaneously detect neonicotinoids such as imidacloprid, dinotefuran, and acetamiprid. OBP2 was selected for its high binding affinity toward these pesticides, enabling the sensor to achieve LOD significantly lower than the international maximum residue levels (MRLs): 1.4 ppb for imidacloprid, 1.5 ppb for acetamiprid, and 4.5 ppb for dinotefuran. To validate the sensor performance, real samples of green and black tea were analyzed, demonstrating a null false‐negative rate (100% true negatives) and a true‐positive rate of 88%. The integration of DiNM technology with OBP2 allows for rapid on‐site screening to identify potential neonicotinoid contamination. When the sensor detects a positive signal, above the cut‐off value, the sample is sent for more detailed and precise analysis via liquid chromatography to confirm the identity and quantify the detected pesticide. This methodology significantly improves the on‐site control process, enabling rapid contamination identification and reducing the workload of standard testing laboratories. The system represents an efficient and sustainable solution for monitoring both food safety and environmental health [180].

In the food sector, in addition to the detection of contaminants, the monitoring of decomposition processes plays a crucial role in ensuring quality and safety throughout the supply chain. A recent study explored the potential of recombinant OBPs from *Hermetia illucens* as bio‐recognition elements in a nanobiosensor for the detection of VOCs indicative of the early stages of organic decomposition, including carboxylic acids, alkanes, ketones, alcohols, aldehydes, amines, and sulfur compounds. The researchers developed a biosensor based on a QCM, where the OBPs from *H. illucens* were immobilized on QCM transducers using SAM. Four OBPs (HillOBP_C57, HillOBP_C11107, HillOBP_C21691, and HillOBP_C1173) were initially selected, among which HillOBP_C57 showed the highest binding affinity toward specific compounds such as isobutyraldehyde, 2‐methylbutyraldehyde, isovaleraldehyde, and butyric acid, typically emitted during the decomposition of animal and plant material. The LOD for the most significant VOCs measured by the sensor based on HillOBP_C57 proved to be remarkably low, highlighting its high sensitivity with values of 0.013 ppm for isobutyraldehyde, 0.004 ppm for isovaleraldehyde, and 0.024 ppm for 2‐methylbutyraldehyde [68]. The use of OBPs, thanks to their high thermal and chemical stability, enabled accurate VOC detection at room temperature, demonstrating the system great potential for real‐time monitoring of organic decomposition processes in environmental and agri‐food contexts.

### 4.5 Insect Pest Control

An olfactory biosensor leveraging OBPs was developed to specifically detect semiochemicals released by insect host plants, facilitating molecular‐level investigation of these interactions. This strategy is pivotal for advancing sustainable, targeted pest management solutions. The biosensor employed BdorOBP2, an OBP derived from the oriental fruit fly ( *Bactrocera dorsalis* ), which was genetically cloned, expressed, and purified before being immobilized onto interdigitated electrodes coated with polyethylene glycol (PEG). This setup formed an (EIS) biosensor.

The interdigitated electrode design enabled accurate and rapid detection of impedance changes, essential for identifying trace levels of specific compounds. The biosensor was evaluated using three semiochemicals, isoamyl acetate, β‐ionone, and benzaldehyde, all emitted by host plants and integral to the fruit fly's chemical communication. Findings revealed that BdorOBP2 exhibited strong binding affinity for these molecules, with LOD ranging from 10^−7^ to 10^−8^ M [181].

This research highlights how insect OBPs, thanks to their stability and selectivity, can be used to develop biosensors that mimic natural olfactory sensitivity. Originally designed to study semiochemicals, these biosensors provide a platform for better understanding chemical interactions between insects and their host plants, allowing for precise detection of compounds that attract insects to specific environments. This capability could support indirect pest control strategies, such as using volatile compounds to lure insects into traps or specific areas, thereby contributing to an ecological approach for pest management [181, 182].

### 4.6 Diagnosis

In medical diagnostics, the ability of OBPs to selectively bind VOCs associated with specific diseases offers a promising foundation for the development of rapid, low‐cost, and non‐invasive detection devices.

The chemical profiles of VOCs undergo significant changes in the presence of diseases, offering a valuable opportunity for early diagnosis and health monitoring [14, 16, 20]. Animals such as dogs and certain insects have demonstrated the ability to detect variations in VOCs associated with diseases, such as cancers and infections, by recognizing changes in odor profiles [14, 131, 132, 133, 134, 135, 136, 183]. These examples highlight the potential of living organisms to discriminate specific VOCs and biomarkers, inspiring the development of biosensors based on OBPs for rapid, non‐invasive, and highly selective diagnostic applications.

Larisika et al. developed a FET sensor based on reduced graphene oxide (rGO), functionalized with the OBP14 protein from the honeybee *A. mellifera* , for detecting homovanillic acid in solution. This compound is of interest as a tumor marker for neuroblastoma and malignant pheochromocytoma and acts as a natural odorant for honeybees. The FET device exhibited good ligand detection performance, with OBP14 immobilized using the PBSE linker, which forms a covalent bond with the rGO surface. Experiments confirmed that binding between the protein and homovanillic acid causes a measurable change in the device source‐drain current, enabling quantitative and specific ligand detection. Kinetic and titration analyses, performed through real‐time current change measurements, showed a binding behavior consistent with the Langmuir model for ligand–receptor interactions. The device demonstrated good selectivity toward different odorants and was able to discriminate ligands with dissociation constants ranging from K_d_ = 4 μM to K_d_ = 3.3 mM. These results indicate the sensors potential as a detection device for environmental and healthcare applications, thanks to its ability to monitor specific biomarkers and chemicals in solution [184, 185].

An alternative to the direct use of OBPs in biosensors is the employment of peptides derived from them. This approach retains the VOC‐binding specificity of OBPs while reducing limitations associated with their molecular size, which can affect the density of binding events in sensitive devices such as CNT‐FET [186]. For example, a biomimetic multiplex sensor was developed based on CNT‐FETs functionalized with peptides derived from olfactory proteins of insects, designed for the selective detection of VOCs associated with COVID‐19. Initially, 10 proteins were selected based on their affinity for VOCs linked to COVID‐19. The VOC‐binding residues extracted from these olfactory proteins were integrated into chimeric peptides containing domains for anchoring to the graphite substrates of CNT‐FETs. Among these, two peptides were synthesized and experimentally tested on CNT‐FETs: MmedPep1‐GrBP, derived from the CSP3 of *Microplitis mediator* (MmedCSP3), and BhorPep1‐GrBP, derived from the OBP of *Batocera horsfieldi* (BhorOBPm2). The tests demonstrated that MmedPep1‐GrBP exhibited a strong affinity for ethyl butyrate, with a current increase of 1.21 ± 0.43 μA, while BhorPep1‐GrBP, sensitive to aldehydes, generated a signal of 0.20 **±** 0.09 μA. The other eight peptides derived from the selected OBPs have not yet been tested on CNT‐FET devices and will be the focus of future studies.

Another example of a peptide‐based biosensor is a QCM device that employs a peptide mimicking the aldehyde‐binding site of HarmOBP7, a protein expressed in the antennae of the moth *Helicoverpa armigera*, for acetaldehyde detection. This peptide was used as a sensing material coated on a polished gold piezoelectric crystal surface. Formation of a thin film by drop‐casting the peptide solution demonstrated sensitivity to acetaldehyde vapor, resulting in the largest shift in the QCM resonance frequency. Specifically, the peptide‐based QCM sensor showed a LOD of 1 ppm at room temperature with 2% ± 1% relative humidity, which is relatively higher sensitivity compared to MOF‐based sensors developed for acetaldehyde [187]. For instance, the PdO‐ZnO p‐n heterojunction nanostructure sensor developed by Majhi et al. showed sensitivity for 100 ppm acetaldehyde at 350°C [188]. Similarly, the Co‐doped ZnO sensor, developed by Shalini and Balamurugan, detects acetaldehyde with a range of 10–500 ppm [189]. These preliminary results demonstrate the effectiveness of biomimetic design, with significant potential for the development of rapid, non‐invasive, and highly selective diagnostic sensors [186].

## 5 Conclusions

OBPs are emerging as excellent candidates for the development of highly sensitive and specific biosensors. Thanks to their intrinsic stability, their ability to function under a wide range of environmental conditions, and their remarkable flexibility in binding to a vast array of VOCs, these systems hold great promise for various applications, including medical diagnostics, environmental monitoring, food safety, and pest control.OBP‐based biosensors offer significant advantages over traditional methods such as GC/MS. While GC/MS provides detailed and highly accurate multi‐component analysis, it also has notable drawbacks: it is bulky, expensive, time‐consuming, and requires highly trained personnel. In contrast, OBP‐based biosensors are portable, rapid, and user‐friendly. These devices can be easily deployed in the field without the need for sophisticated infrastructure or labor‐intensive sample preparation, enabling cost‐effective and immediate detection, ideal for practical applications in extreme conditions or remote locations. A further advantage of insect OBPs lies in their sustainability, as they can be produced through biotechnological methods, such as heterologous expression in different systems, using renewable biological resources and reducing the need for complex synthetic materials. Furthermore, OBP‐based biosensors do not require toxic solvents, unlike conventional systems, thereby contributing to the reduction of environmental impact and chemical pollution. An additional advantage of OBPs as recognition elements is their high versatility: they can be used to detect ligands in aqueous phase or volatile phase. Their ability to reversibly bind a wide range of molecules makes them suitable for various sensing applications. Depending on the specific requirements of the intended application, OBPs can be integrated with different transduction platforms, including electrochemical, optical, and piezoelectric sensors. Despite the progress made, the practical application of these devices requires further advancements. Methods for immobilizing OBPs on sensor substrates need to be optimized, specificity in complex environments must be enhanced, and long‐term stability should be ensured. Additionally, scalability and cost‐effectiveness remain critical challenges for the widespread adoption of these technologies. Integration with advanced technologies, such as nano‐bioelectronics and artificial intelligence, represents a key direction for improving data processing and interpretation capabilities. Future research should focus on developing sensors capable of detecting multiple analytes simultaneously, engineering OBPs to improve their binding properties, and analyzing their performance under extreme conditions. The favorable comparison with traditional technologies like GC/MS underscores the vast potential of OBP‐based biosensors to meet the growing needs of sectors such as healthcare, agriculture, and environmental control, offering sustainable, high‐performing, and accessible solutions.

## 6 Future Directions

In the near future, the use of OBPs could evolve from simple molecular recognition elements to key components of bio‐digital tools capable of processing chemical information in a dynamic and contextual way. It will no longer be a matter of simply detecting a ligand, but of interpreting a real chemical language, recognizing distinctive patterns and combinations of complex biological or environmental states. However, this transition requires addressing some already known critical issues, including the complexity of the olfactory code and the difficulty of artificially reproducing the extremely high sensitivity and selectivity of biological systems; the management of interference generated by environmental factors, such as humidity or high concentrations of analytes; and, finally, the persistent lack of standardization and shared regulations, which limits the validation, comparison, and industrial scalability of OBP‐based platforms. The integration of OBP‐based systems, innovative materials, and machine learning algorithms will enable the creation of intelligent olfactory platforms inspired by the logic of living organisms. In this perspective, OBPs become the bridge between biology and technology.

## Author Contributions

Conceptualization: Patrizia Falabella. Writing, original draft preparation: Patrizia Falabella, Maddalena Ventura, Miriam Viola, Carmen Scieuzo. Writing, review and editing: Patrizia Falabella, Maddalena Ventura, Miriam Viola, Krishna C. Persaud, Antonio Guerrieri, Carmen Scieuzo. Supervision: Patrizia Falabella, Carmen Scieuzo.

## Funding

This work was supported by the University of Basilicata and by the Italian Ministry of University and Research under Decree No. M 117/2023, through a Ph.D. scholarship co‐financed by Sharing Communications Agency Srl.

## Conflicts of Interest

The authors declare no conflicts of interest.

## Data Availability

Data sharing not applicable to this article as no datasets were generated or analyzed during the current study.
